# Ferroptosis-related genes, a novel therapeutic target for focal segmental glomerulosclerosis

**DOI:** 10.1186/s12882-024-03490-5

**Published:** 2024-02-17

**Authors:** Yanbin Lin, Jinxuan He, Zhixiang Mou, Huiting Chen, Wenkang You, Tianjun Guan, Lan Chen

**Affiliations:** 1 Department of Nephrology, Zhongshan Hospital of Xiamen University, School of Medicine, Xiamen University, Xiamen, China; 2 Fujian Medical University, Fuzhou, China; 3 Xiamen Municipal Health Commission, Xiamen, China

**Keywords:** Bioinformatics, Ferroptosis, Focal segmental glomerulosclerosis (FSGS), Renal tubule

## Abstract

Recent studies have suggested that ferroptosis participates in various renal diseases. However, its effect on focal segmental glomerulosclerosis remains unclear. This study analyzed the GSE125779 and GSE121211 datasets to identify the differentially expressed genes (DEGs) in renal tubular samples with and without FSGS. The Cytoscape was used to construct the protein–protein interaction network. Moreover, the ferroptosis-related genes (FRGs) were obtained from the ferroptosis database, while ferroptosis-related DEGs were obtained by intersection with DEGs. The target genes were analyzed using Gene Ontology (GO) and Kyoto Encyclopedia of Genes and Genomes (KEGG) analysis. The GSE108112 dataset was used to verify the expression of target FRGs. Besides, we built the mRNA-miRNA network regarding FRGs using the NetworkAnalyst database, and circRNAs corresponding to key miRNAs were predicted in the ENCORI database. In this study, 16 ferroptosis-related DEGs were identified between FSGS and healthy subjects, while five co-expressed genes were obtained by three topological algorithms in Cytoscape. These included the most concerned Hub genes JUN, HIF1A, ALB, DUSP1 and ATF3. The KEGG enrichment analysis indicated that FRGs were associated with mitophagy, renal cell carcinoma, and metabolic pathways. Simultaneously, the co-expressed hub genes were analyzed to construct the mRNA-miRNA interaction network and important miRNAs such as hsa-mir-155-5p, hsa-mir-1-3p, and hsa-mir-124-3p were obtained. Finally, 75 drugs targeting 54 important circRNAs and FRGs were predicted. This study identified the Hub FRGs and transcriptomic molecules from FSGS in renal tubules, thus providing novel diagnostic and therapeutic targets for FSGS.

**Supplementary Information:**

The online version contains supplementary material available at 10.1186/s12882-024-03490-5.

## Introduction

Focal segmental glomerulosclerosis (FSGS) is characterized by partial glomerular (focal) or partial glomerular capillary loops (segmental) sclerosis. In most parts of the world, FSGS is one of the leading causes of glomerulopathy in end-stage renal disease (ESRD) [1–3]. The treatment of FSGS mainly includes corticosteroids, immunosuppressive therapy, and kidney transplantation. Unfortunately, FSGS patients have become increasingly resistant to corticosteroids and immunosuppressive therapy, thus showing a limited response to FSGS. The existing treatment modalities and efficacy of FSGS are insufficient in delaying the progression of ESRD, which necessitates more effective treatments for the management of FSGS [4–7]. The study reported that the degree of tubular injury and interstitial lesions in FSGS patients were related to the degree and extent of glomerular involvement. With the progression of glomerular disease, tubular atrophy and interstitial fibrosis increase. However, there is a lack of studies on the expression of tubular genes in diseased kidneys.

Ferroptosis was first proposed by Dr. Brent R. Tockwell in 2012. It is defined as a new programmed cell death procedure characterized by the accumulation of reactive oxygen species and the overload of intracellular ions, thus causing lipid peroxidation [8]. Its mechanism largely involves the upregulation of unsaturated fatty acids (USFAs) on cell membranes based on ester oxygenase and divalent ion effect, while USFAs can be catalyzed to produce lipid peroxidation, eventually causing cell apoptosis. Ferroptosis takes place during various chronic disorders, including renal cancer, pancreatic cancer, Parkinson's syndrome, and cardiomyopathy [9]. The involvement of ferroptosis in acute kidney injury (AKI) has been reported in nephrology but not in FSGS.

Previous studies have reported that ferroptosis does not have the morphological characteristics of traditional apoptosis, such as cell wrinkling, chromatin agglutination, and apoptotic body formation, although the characteristic changes such as mitochondrial wrinkling and increased bilayer density can be observed by electron microscopy. Studies on the model of FSGS induced by Adriamycin amycin (ADR) in mice have reported changes such as mitochondrial atrophy, reduction in mitochondrial crista, and rupture of the mitochondrial membrane in the podocytes of mice models of FSGS, suggesting that the mitochondria of FSGS podocytes have morphological changes that characterize ferroptosis [10]. Recent studies have confirmed that both GSH depletion and lipid peroxide accumulation are characteristic changes of ferric death [11]. Decreased GSH will impact the glutathione peroxidase activity, decrease the cell antioxidant ability, and promote lipid ROS and lipid peroxidation, resulting in ferric death [12]. The concentration of GSH in the kidney tissue of the above FSGS model mice was significantly decreased, while the concentration of MDA, a product of lipid peroxidation, was significantly increased, indicating the occurrence of ferric death in FSGS. In 2022, Vanswelm et al. published a study suggesting that the accumulation of renal iron in experimental FSGS was associated with the progression of tubulointerstitial (TI) injury in FSGS models. Besides, the reduced renal iron load avoided TI fibrosis [12]. This offers a specific method for the reduction of renal iron load while maintaining the minimal cell iron level required to maintain an optimal balance of renal iron. Thus, the mechanism of ferric death in FSGS is still unclear, with studies focusing on renal tubular tissue in FSGS being rare. Therefore, the study of differential genes involved in renal tubular lesions in FSGS and the interaction between them will improve understanding of this field.

In this study, the DEGs of the two datasets were retrieved through the GEO database, and the rich biological functions and pathway information of the differential genes were obtained through the Gene Ontology (GO) enrichment, Kyoto Encyclopedia of Genes and Genomes (KEGG) pathway enrichment. Then, the ferroptosis-related genes (FRGs) in FSGS were obtained by a Venn diagram, and incorporated into the STRING database to construct a corresponding protein–protein interaction (PPI) network. Thereafter, the five most significantly correlated genes were chosen as the key genes using the CytoHubba plugin. The GSE108112 dataset was then used to assess the expression of these genes. The mRNA-miRNA network was constructed based on the five central genes to explore the possible regulation of miRNAs on FRGs in FSGS and explore the potential circRNAs and target drugs.

## Methods

### Data acquisition

Data were obtained from the mRNA expression profile datasets GSE121211, GSE125779, and GSE108112 from the GEO database (https://www.ncbi.nlm.nih.gov/geo/) [13]. The research object was *Homo sapiens*, "focal segmental glomurular sclerosis and FSGS" were used as the keyword, and the research content included renal tubular tissue. Based on the FerrDb database (http://www.zhounan.org/ferrdb/), 259 genes were observed to be associated with ferroptosis.

### Screening for differential genes associated with ferroptosis

The gene expression profiles between the normal and disease groups were obtained from the GEO database. The data provided by the original submits were extracted using the GEO query R package. The value distribution of the selected samples was visualized using an R boxplot algorithm. The limma R package was utilized for performing differential analysis of the data to identify differential genes. DEGs from the two datasets were obtained based on the criteria of *P* < 0.05 and |logFC|≥ 0.5, and genes associated with ferroptosis were identified according to their differential expression between the normal and disease groups and through the FerrDb database and termed as ferroptosis-related genes.

### GO functional enrichment analysis and KEGG pathway enrichment analysis of differential genes

The GO database (https://geneontology.org) [14] was established by the Gene Ontology Consortium and included biological processes (BP), molecular functions (MF), and cellular components (CC). The KEGG Database (https://www.kegg.jp/) [15] is a database that integrates information on the genomic, chemical, and systematic functions.

### Construction of PPI network and module analysis

The STRING database (https://string-db.org/) was used to analyze the PPI network for DEGs [16]. In addition to exploring the relationships among the DEGs, a confidence score > 0.7 was set as significant. Then, the software Cytoscape (version 3.9.0) was employed to import the results of the STRING analysis. The top five most significant genes were selected as the hub genes based on MCC, degree, and MNC algorithm, using the CytoHubba plugin of Cytoscape [17].

### Construction of an mRNA-miRNA regulatory network

The NetworkAnalyst3.0 database (https://www.networkanalyst.ca/) was used to predict the associations of differentially expressed mRNAs (DEmRNAs) with differentially expressed miRNAs (DEmiRNAs) [18]. Then, the obtained data were imported into Cytoscape, the mRNA-miRNA regulatory network was built to describe the miRNA-mRNA interactions as the possible targets for tubular tissues from kidneys with FSGS. We then visualized the network using the Cytoscape software.

### Screening of circRNAs corresponding to miRNAs

The online database ENCORI (https://starbase.sysu.edu.cn/) [19] was used to predict the interactions between differential expression of miRNA and circRNA. The circRNAs corresponding to the previously predicted key miRNAs are presented as flower plot.

### Validation of ferroptosis hub genes

The programs “ggplot2 and pROC” (R3.6.3) were used to perform the receiver operating characteristic (ROC) analysis to determine the specificity and sensitivity of target genes. Then, the area under the ROC curve (AUC) value was determined to quantify the results. Genes showing an AUC value of > 0.6 were identified as the diagnostic genes.

### Prediction of potential therapeutic drugs

Genes from the DSigDB database (http://tanlab.ucdenver.edu/DSigDB) drug interaction data used to predict the potential therapeutic drugs for FSGS.

## Results

### Screening for differential genes

**Fig. 1:**
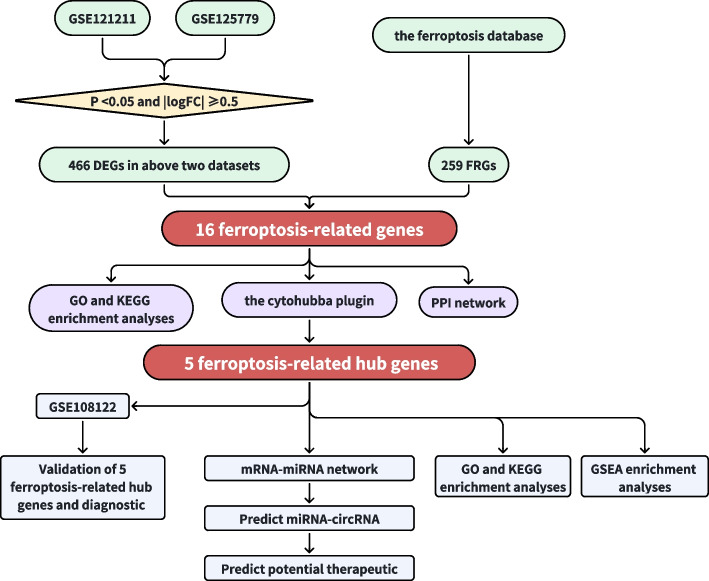
The overall protocol of this study. Abbreviations: *DEGs* Differentially expressed genes, *FSGS* Focal segmental glomerulosclerosis, *GO* Gene Ontology, *KEGG* Kyoto Encyclopedia of Genes and Genomes, *FRGs* Ferroptosis-related genes

**Fig. 2:**
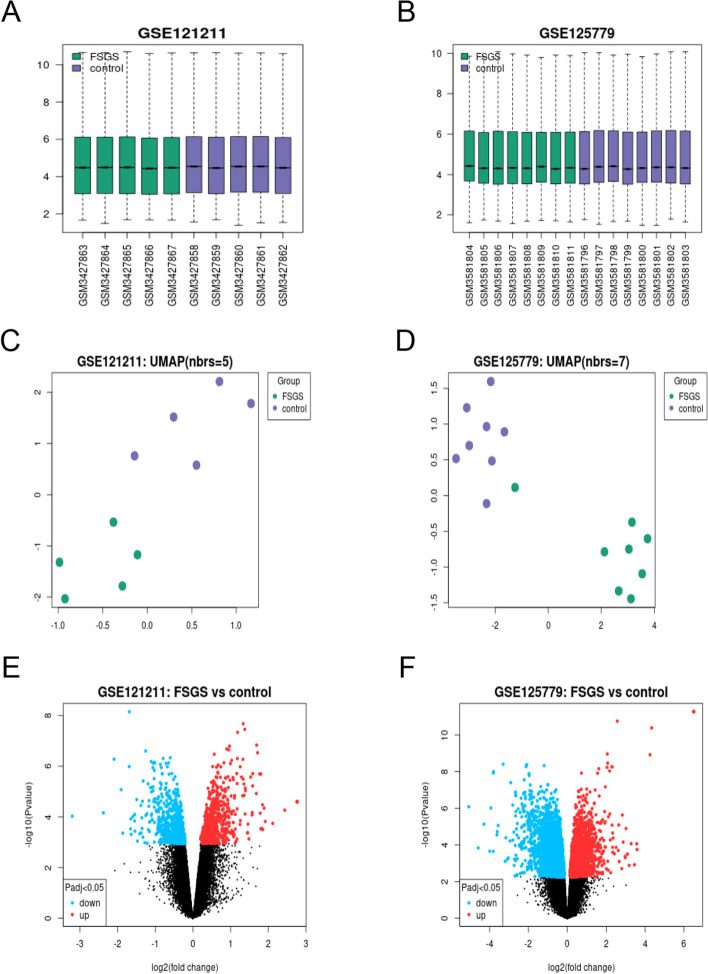
Datasets analysis of GSE121211 and GSE125779. **A**-**B** Normalized expression matrices, (**C**-**D**) UMAP analysis and (**E**–**F**) Volcano plot of the GSE121211 and GSE125779. Each point on the volcano map represents a gene. Blue indicates a down-regulated gene, and red indicates an up-regulation gene

**Fig. 3:**
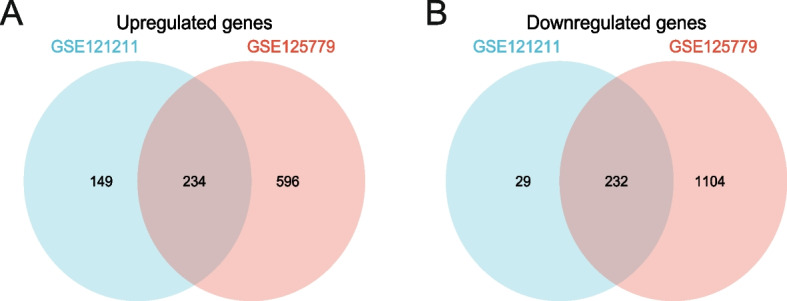
Venn diagram of DEGs common to two GEO datasets. **A** A total of 446 consistently expressed genes were identifified from GSE125779 and GSE121211, including 234 upregulated genes and (**B**) 232 downregulated genes in FSGS kidney tissues compared to the control. Different color areas represented different datasets. The cross areas indicate the common expressed genes

The GSE121211 dataset contains five FSGS samples and five normal samples, while the GSE15779 dataset contains eight FSGS and eight corresponding normal specimens (Fig. 1). After normalization of the expression matrices of the two datasets, the box plot distribution trend revealed nearly straight lines (Fig. 2A and B). To assess the in-group data reproducibility, UMAP analysis was performed on the two datasets, which showed good reproducibility (Fig. 2C and D). Through the “limma” package of the R language, the analysis of variance between the two datasets (screening criteria were *P* < 0.05 and |logFC|≥ 0.5) revealed upregulated and downregulated DEGs from the GSE121211 dataset and GSE125779 dataset (Fig. 2E and F). A total of 234 upregulated and 232 downregulated DEGs were obtained (Fig. 3A and B). The specific 466 DEGs are detailed in Additional file 1.

### Ferroptosis-related genes in FSGS

**Fig. 4:**
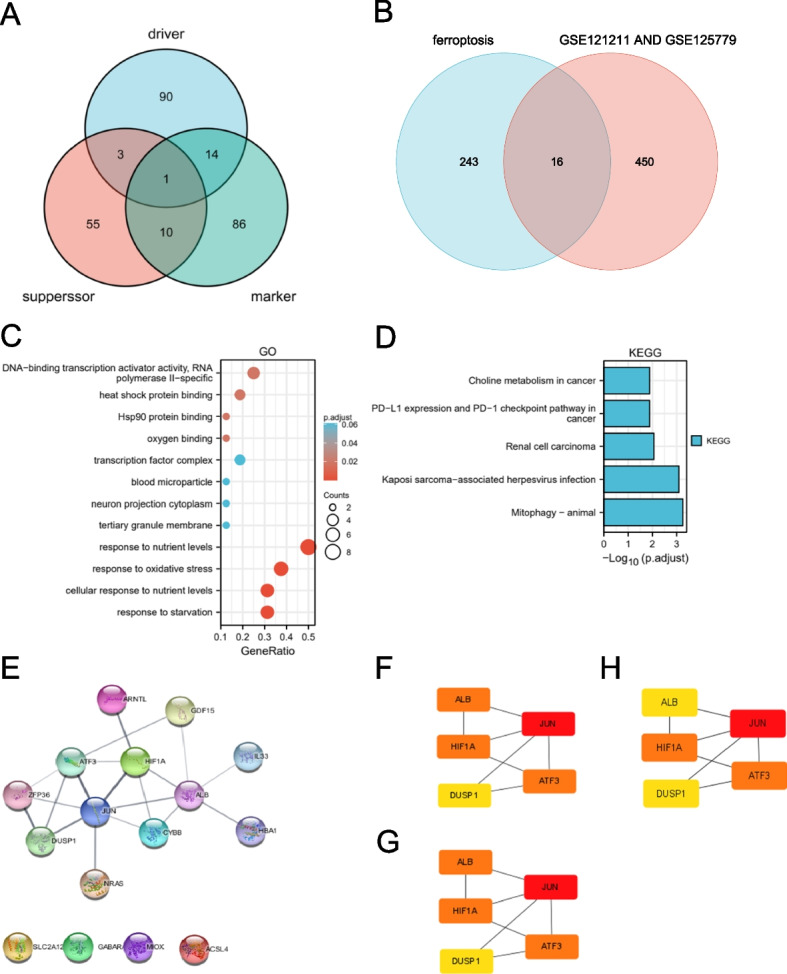
Ferroptosis-related genes analyses. **A** 259 FRGs in the ferroptosis database. **B** Venn diagram of FRGs to two GEO datasets and the ferroptosis database. **C** GO enrichment analysis of 16 FRGs in FSGS. **D** KEGG enrichment analysis of 16 FRGs in FSGS. **E** PPI networks of 16 FRGs in FSGS. **F**–**H** Top5 FRGs in the PPI works

A total of 259 FRGs were obtained from the ferroptosis database, including 108 genes with driver function, 69 genes with suppressor function, 111 genes with marker function, and one gene with three different roles (HMOX1) (Fig. 4A). When the two-cube variance analysis of the datasets GSE121211 and GSE125779 was set to the standard *P* < 0.05 and |logFC|≥ 0.5, 466 different genes and 16 ferroptosis-related genes were obtained with the combination of the related database (Fig. 4B). The GO functional enrichment analysis of 16 ferroptosis-related genes and KEGG pathway enrichment analysis results in Fig. 4C and D. The results of the PPI network analysis are presented in Fig. 4E. Interaction maps of important proteins were obtained by the software Cytoscape, with a total of 19 edges and 12 nodes, with one edge representing protein interaction and one node representing one protein. The node size was adjusted according to the degree. PPI results were imported into Cytoscape and opened with the CytoHubba plugin. The top five genes of MCC, MNC, and degree topology algorithms were selected to obtain common genes as ferroptosis-related hub genes (Fig. 4F-H). A total of five hub genes were obtained, which were JUN, ALB, ATF3, HIF1A, and DUSP1 (Table 1).

**Table 1:** Five hub genes and their functions

Gene symbol	Description
JUN	Jun proto-oncogene, AP-1 transcription factor subunit
ALB	albumin
ATF3	activating transcription factor 3
HIF1A	hypoxia inducible factor 1 subunit alpha
DUSP1	Dual specificity phosphatase 1

### Gene network establishment and GO/KEGG analysis of FRGs

**Fig. 5:**
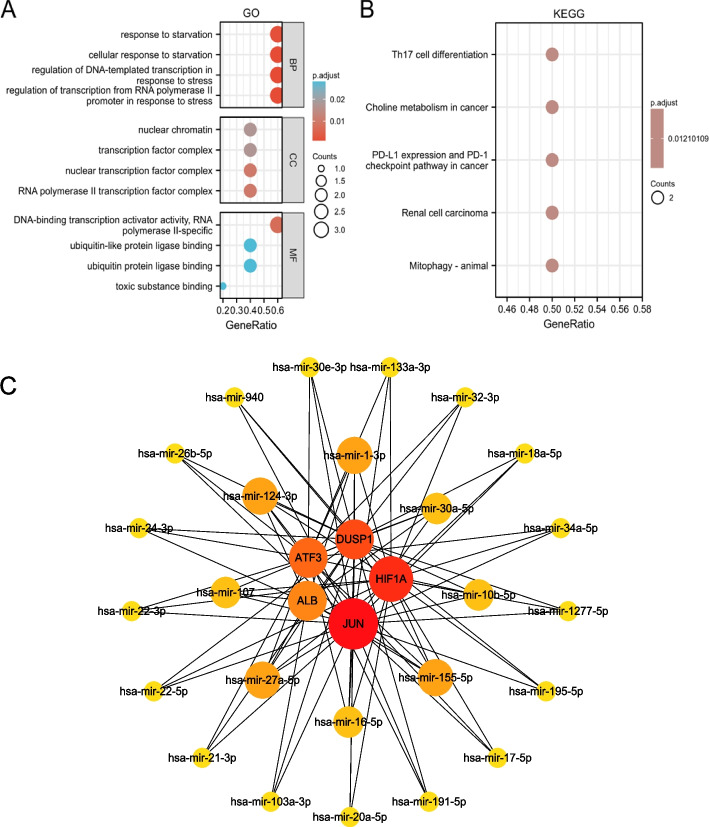
mRNA-miRNA regulatory network and GO/KEGG of 5 ferroptosis-related hub genes. **A**-**B** GO/KEGG categories and pathways. **C** mRNA-miRNA regulatory network. All four miRNAs hsa-mir-155-5p, hsa-mir-124-3p, hsa-mir-27a-5p, and hsa- mir-1-3p were correlated with the five specifically expressed FRGs, which is highly significant. Abbreviations: *BP* Biological processes, *CC* Cellular components, *MF* Molecular function

**Fig. 6:**
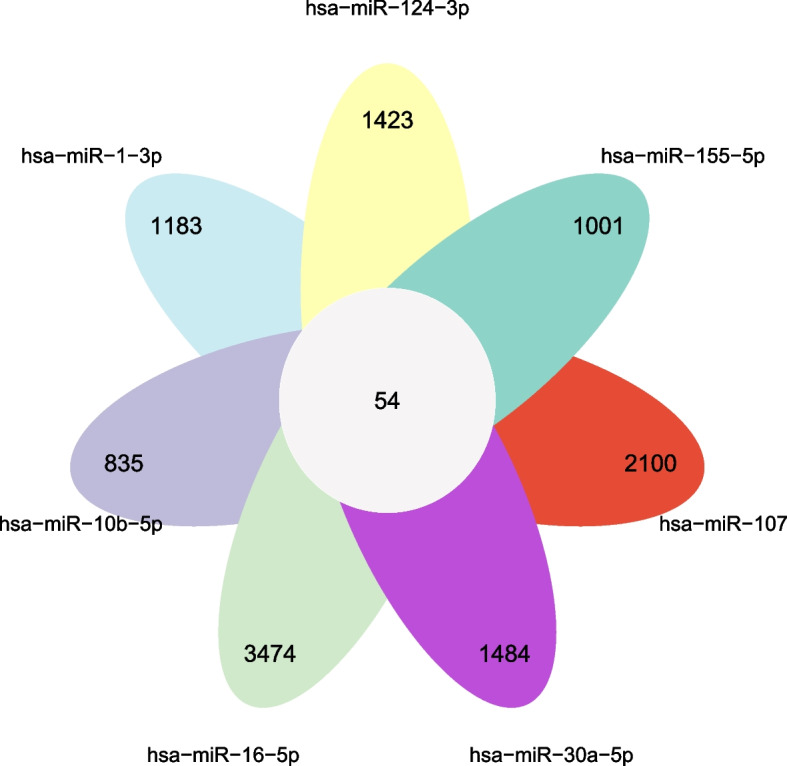
The flower plot showed that seven circRNAs corresponding to miRNAs were screened in the ENCORI database

The program NetworkAnalyst 3.0 was used to predict the target miRNAs for key genes. Finally, 240 target miRNAs for five specific FRGs were obtained, and 333 mRNA-miRNA pairs were determined. A total of 118 miRNAs regulated JUN, 101 regulated HIF1A, 79 regulated DUSP1, nine modulated ALB, and 26 modulated ATF3. All four miRNAs hsa-mir-155-5p, hsa-mir-124-3p, hsa-mir-27a-5p, and hsa-mir-1-3p were correlated with the five specifically expressed FRGs, which is highly significant. Each of the four miRNAs, hsa-mir-6-5p, hsa-mir-30a-5p, hsa-mir-107, and hsa-mir-10b-5p had four genes associated with them (Fig. 5). The miRNAs are shown in additional file 2. CircRNAs corresponding to hub miRNAs were screened in the ENCORI database (no corresponding circRNA was predicted in the ENCORI database for hsa-mir-27a-5p), and a total of 54 common associated circRNAs were obtained (Fig. 6). The specific circRNA information is detailed in additional file 3.

The five ferroptosis-related DEGs were subjected to GO and KEGG analysis. The most significantly enriched GO terms included transcription regulation of RNA polymerase II promoter upon stress, regulation of DNA-templated transcription upon stress, response to starvation, nuclear transcription factor complex, RNA polymerase II transcription factor complex, the activity of DNA-binding transcription activator, transcription factor complex, and RNA polymerase II-specific and nuclear chromatin. The results of KEGG indicated that these DEGs were mostly associated with mitophagy-animal, renal cell carcinoma, choline metabolism, Th17 cell differentiation, PD-L1, expression, and PD-1 checkpoint pathway in cancer (Fig. 5A and B).

### GSE108112 confirmed the expression and diagnostic value of FRGs

**Fig. 7:**
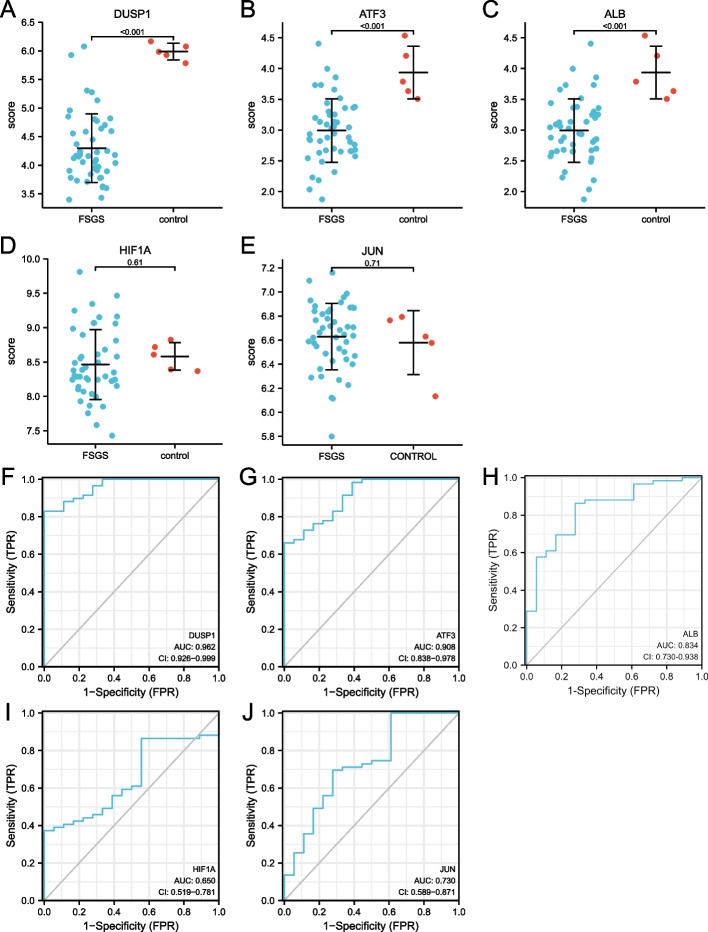
Comparison of the expression and diagnostic ROC curves of 5 ferroptosisrelated hub genes. **A**-**E** Comparison of the expression of 5 ferroptosis related hub genes in FSGS and healthy samples. **F**-**J** Diagnostic ROC curves of 5 ferroptosis related hub genes in FSGS and healthy samples. *ROC* Receiver operating characteristic, *TPR* True positive rate, *FPR* False positive rate

The dataset GSE108112 was used to check the levels of selected targets. It was observed that three key FRGs (ALB, ATF3, and DUSP1), which were differentially expressed between renal tubular tissues with and without FSGS in patients receiving tumor nephrectomy, conformed to the predicted results (Fig. 7A-E).

**Fig. 8:**
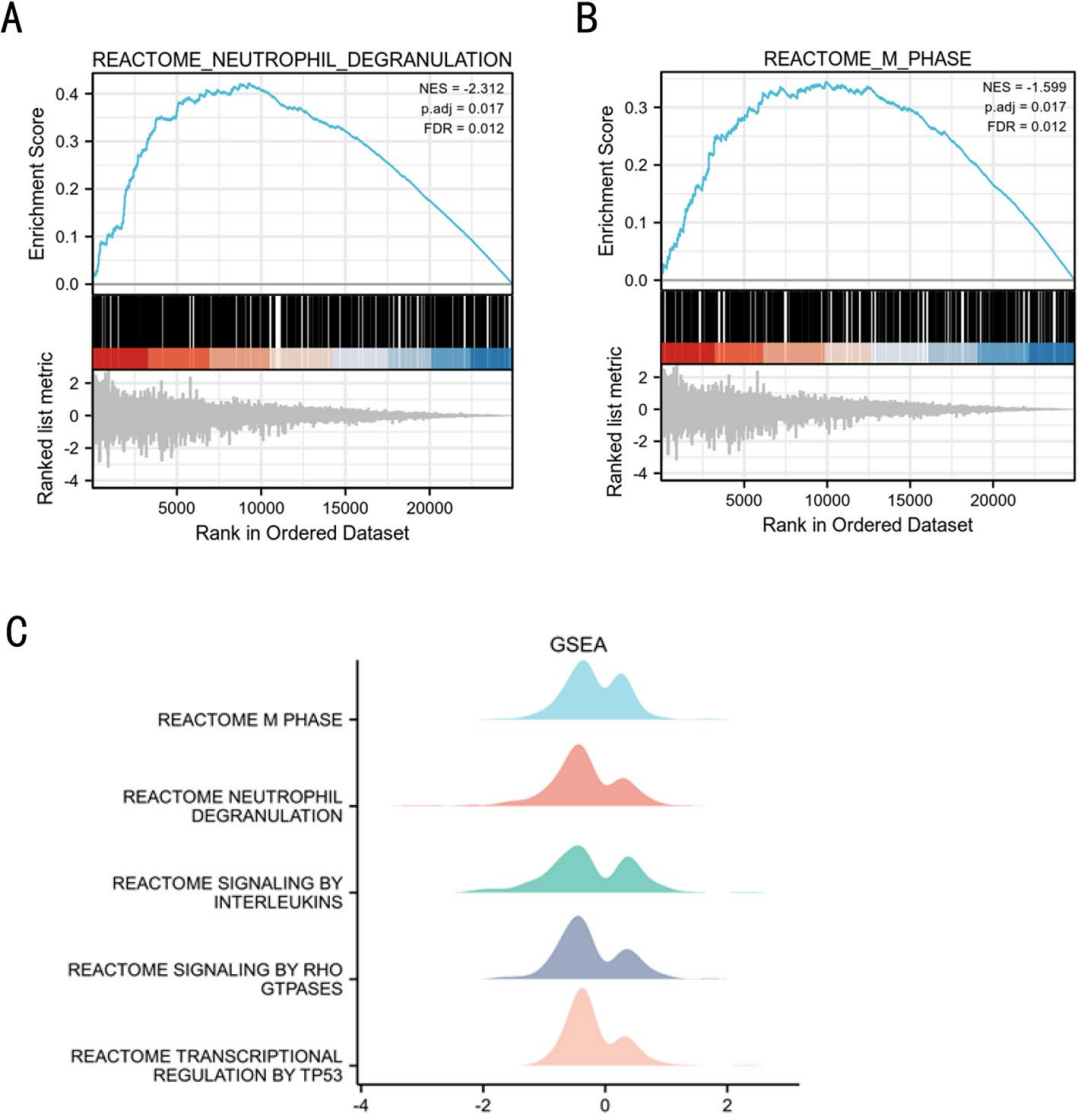
Gene set enrichment analysis. GSEA in FSGS. NOM p-val < 0.05, FDR < 25%. **A**-**B** signaling pathways where the 5 ferroptosis-related hub genes are predominant in FSGS and healthy samples. **C** Specific signal pathway enrichment of 5 ferroptosis-related hub genes

Then, the GSEA approach was used for functional enrichment between FSGS and normal subjects, and the obtained results were compared with the five above-mentioned genes. It was observed that the genes were mainly present in the REACTOME_M_PHASE (NES = -1.599; P.adjust = 0.017; FDR = 0.012) and REACTOME_NEUTROPHIL_DEGRANULATION (NES = -2.312; P.adjust = 0.017; FDR = 0.012) signal pathway enrichment (Fig. 8A-C).

The ROC curves were plotted based on the obtained data in renal tubular tissues with and without FSGS. The above five genes were identified as the diagnostic genes for FSGS. The AUC value of the variable DUSP1 was 0.962 (95% CI: 0.926–0.999), while the AUC values for ATF3, ALB, JUN, and HIF1A were 0.908 (95% CI: 0.0838–0.978), 0.834 (95% CI: 0.730–0.938), 0.730 (95% CI: 0.589–0.0.871), and 0.650 (95% CI: 0.519–0.781), respectively (Fig. 7F-J).

### Targeted drug prediction

We used the DSigDB database to predict the potential targeted agents that were associated with FRGs, which might treat FSGS by modulating ferroptosis. A total of 75 drugs were predicted, among which, nitroglycerin was identified as a targeted inhibitor of HIF1A (Table 2).

**Table 2:** Drug prediction of ferroptosisrelated genes

Gene symbol	drug
JUN	SERTRALINE、MECHLORETHAMINE HYDROCHLORIDE、TROPISETRON、BUPROPION HYDROCHLORIDE、AZELASTINE HYDROCHLORIDE、CUPRIC CHLORIDE、CIPROFIBRATE、FENOFIBRATE、VINBLASTINE SULFATE、ATOMOXETINE、HYDROCHLORIDE、CINNARIZINE、COLCHICINE、DIPHENHYDRAMINE、HYDROCHLORIDE、QUINAPRIL、HYDROCHLORIDE、CLOFIBRATE、TRIFLUPROMAZINE、HYDROCHLORIDE、GEMFIBROZIL、CLOTRIMAZOLE、VINORELBINE TARTRATE、METHIMAZOLE
ALB	PYROGALLOL、IODIPAMIDE、DIAZEPAM、DICLOFENAC、OLMESARTAN MEDOXOMIL、RALTITREXED、NAPROXEN、WARFARIN、FLUCONAZOLE、GADOFOSVESET
ATF3	PROGESTERONE
HIF1A	PHENOXYBENZAMINE HYDROCHLORIDE、NIFEDIPINE、PIRETANIDE、EPINEPHRINE、EPINEPHRINE BITARTRATE、AXITINIB、TRIAMTERENE、DEQUALINIUM、BENZBROMARONE、VINCRISTINE SULFATE、INAMRINONE、NITROGLYCERIN、AMCINONIDE、TRETINOIN、DESOXIMETASONE、CYCLOSERINE、OXYTETRACYCLINE、DICLOFENAC SODIUM、NICLOSAMIDE、ISOETHARINE MESYLATE、LORATADINE、HYDROQUINONE、OXATOMIDE、ISOPROTERENOL、MEFENAMIC ACID、DOPAMINE、TOLFENAMIC ACID、SULFASALAZINE、EPOETIN ALFA、ETHAMSYLATE、FLUFENAMIC ACID、NOREPINEPHRINE BITARTRATE、CLOTRIMAZOLE、SORAFENIB、PROMAZINE、LEVONORDEFRIN、PIMOZIDE、DEFEROXAMINE、NOSCAPINE、HYDROCORTISONE、TOPOTECAN HYDROCHLORIDE
DUSP1	HYDROXYUREA、ALBUTEROL、VASOPRESSIN

## Discussion

In this study, bioinformatics was used to screen the differential genes related to ferroptosis in the FSGS renal tubule tissue and healthy control datasets, yielding a total of 16 FRGs. By constructing the PPI interaction network, the CytoHubba plugin was used to obtain the five most important ferroptosis-related hub genes, namely JUN, ALB, ATF3, HIF1A, and DUSP1. The related pathways and biological functions were enriched and analyzed to identify the vital functions of these genes in transcriptional regulation. These genes play a significant role in mitochondria and tumor metabolic pathways. By constructing the mRNA-miRNA network of five genes, four miRNAs with the highest degree of relationship were found, namely hsa-mir-155-3p, hsa-mir-124-3p, hsa-mir-27a-5p, and hsa-mir-1-3p. By analyzing the key miRNAs, the corresponding 54 circRNAs and potential disease-targeting drugs such as nitroglycerin to inhibit HIF1A may represent a breakthrough in the treatment of FSGS.

HIF1A, the key gene for ferroptosis that was identified in our study, has been confirmed in acute or chronic kidney injury. In the renal ischemia–reperfusion model, renal tubular epithelial cells and renal microvasculature are damaged, while iron cells can recruit and promote macrophage stimulation for the recruitment of neutrophils, thereby inducing the AKI inflammatory reaction. Importantly, ACSL4 is an important target for the regulation of ferroptosis, and HIF1A can bind to the ACSL4 promoter. In this study, the expression of ACSL4 was negatively regulated, while HIF1A was associated with response to cellular stress in FSGS [20]. In another important study, it was also noted that roxaduxat, an inhibitor of HIF proline hydroxylase, protects renal tubular epithelial cells from iohexanol-induced damage both in vivo and in vitro by stabilizing the HIF1A and activating the downstream BNIP3-mediated mitophagy [21]. Our study also enriched the mitophagy pathway, further demonstrating the important role of HIF1A in renal tubules. Furthermore, Wang et al. confirmed that HIF1A was a tumor suppressor in RCC by mining the ENCORI database and TCGA database, combined with a cohort study by the experimental team. Their study also reported that the Snp-mediated upregulation of lncRNA-ENTPD3-AS1 suppressed renal cell carcinoma (RCC) via the mir-155/HIF1A pathway [22]. This finding was in line with the RCC-related pathways enriched in this study. Therefore, we hypothesized that the pathological mechanism of RCC and FSGS may be somewhat similar, with hsa-mir-155 and HIF1A playing an important role. There is a lack of studies on FSGS and HIF1A and the mechanism needs further exploration.

ALB has been extensively suggested to have a critical effect on the mechanism of ferric death in FSGS and is the key gene of ferric death that was enriched in this study, with a high diagnostic efficiency based on the ROC curve enrichment results. As the most abundant protein gene encoded in human blood, ALB plays an important role in regulating the osmotic pressure of plasma colloids and can be used as a carrier protein for various endogenous molecules such as metabolites and fatty acids, which are related to ferroptosis-related BPs such as lipid metabolism, iron metabolism, and amino acid metabolism [23]. FSGS patients with low serum albumin levels of glomerular pathological damage possibly have proteinuria, decreased serum propagation, increased compensatory synthesis and metabolism of kidney burden of liver cells, which increase hypoalbuminemia, induce infection, cause endocrine disturbance and loss of trace elements, high condensation, and impaired immune function, eventually inducing metabolic disturbance [24]. Presently, bioinformatics has been used to analyze the whole kidney tissue to obtain the related genes involved in tissue fibrosis in the mechanism of FSGS. In this study, renal tubules were directly selected for further analysis, the target genes involved in ferroptosis were obtained, and the expression of differential genes of renal tubular lesions was further elaborated, which provided a new target for more precise treatment.

Although the mechanisms related to ferroptosis in FSGS are still unclear, bioinformatics may open a new world in FSGS research. The regulatory functions of miRNAs, a class of non-coding RNAs, involve their ability to recognize target gene mRNA and guide the silencing complex for either degradation or translational inhibition. The construction of an mRNA-miRNA network can enhance our understanding on their intrinsic regulatory role and provide evidence for future therapeutic interventions. In this study, through the construction of the mRNA-miRNA network, we observed that hsa-mir-124-3p has important research significance and plays a role in enriched tumor pathways [25–29]. In a previous experiment involving an animal model of hypertension, EGR1 was found to be the target gene of hsa-mir-124-3p. In that study, silencing EGR1 could overexpress hsa-mir-124-3p, thereby inhibiting the effect of ANG-II on promoting apoptosis and the production of ROS in HUVEC cells. Simultaneously, the accumulation of ROS is among the important signs of ferroptosis, which also indicates that hsa-mir-124-3p may be involved in ferroptosis and play a role in regulating blood pressure [30]. In a study on renal injury, overexpression of mir-124-3p can partially reverse the inhibition of cell proliferation, induction of apoptosis, and aggravation of cell injury caused by the overexpression of HOXA11-AS, which proves that HOXA11-AS regulates the inflammatory response in calcium oxalate crystal-induced renal injury through the mir-124-3p/McP-1 pathway [31]. This study demonstrates that hsa-mir-124-3p plays an important role in the ferroptosis-related mechanism of FSGS, although further experimental verification is warranted.

In recent years, novel drugs have been developed for the treatment of FSGS, including the B7-1 inhibitor abatacept [32], the latest generation of CD 20 monoclonal antibody (adalimumab) [33], mTOR inhibitor sirolimus [34], and others. According to Anders HJ et al., recent studies have demonstrated that Dapagliflozin is an effective renal protection agent in patients with FSGS nephropathy [35]. Additionally, Sparsentan, a dual receptor angiotensin receptor type 1 and endothelin type A receptor blocker, is the first drug developed and evaluated exclusively for the indication as a treatment for FSGS. Sparsentan has emerged as a promising new treatment option by effectively reducing proteinuria compared to irbesartan and slowing down kidney disease progression [36]. However, there remains an unmet need for FSGS treatment. The study emphasizes the significance of biomarkers and targeted agents based on underlying mechanisms classification and identification of agents capable of preventing or reversing fibrosis processes. This study preliminarily explores potential treatments for FSGS by predicting target drugs using differentially expressed genes associated with core ferroptosis; however, specific treatment options still require further development.

Most of the previous studies on FSGS were single-gene experimental confirmatory studies, focusing more on glomerular damage, with the latest studies using bioinformatics methods focusing only on the whole kidney tissue. However, our study was more accurately focused on the renal tubules, with ferric death as the novel mode of cell death, and used bioinformatics to comprehensively evaluate the effect of multiple genes on FSGS. We identified five potential FRGs (JUN, ALB, ATF3, HIF1A, and DUSP1) with good diagnostic performance. This work built the relevant mRNA-miRNA interaction network to obtain critical miRNAs such as hsa-mir-124-3p and hsa-mir-155-5p, to predict the possible related circRNAs and targeted drugs.

This study also has certain limitations. Although different validation datasets were analyzed together, the results of the analysis were predictive. The study lacks cell experiments, animal experiments, and verification. Although several studies have reported that the concentration of HIF1A in the FSGS group is significantly different from that in other groups, our validation set did not show a similar finding, thus necessitating further experimental verification. Ferroptosis and FSGS are presently poorly studied. In the future, more studies will reveal the molecular mechanisms involved in ferroptosis, which will provide more evidence for ferroptosis in the prevention and treatment of FSGS.

## Conclusion

In summary, this study identified five ferroptosis-related genes, which may be potential novel diagnostic markers for renal tubulointerstitial injury in FSGS. Our prediction of several miRNAs will provide valuable reference information for the ferroptosis-related pathological mechanism of FSGS, and the prediction of targeted drugs provides direction for clinical treatment. Further clinical and basic studies are needed to elucidate the specific mechanistic details of ferroptosis in FSGS-associated tubulointerstitial damage in the future.

### Supplementary Information

**Additional file 1.**

**Additional file 2.**

**Additional file 3.**

## Data Availability

The datasets analyzed by the current study are available in the GEO repository, [https://www.ncbi.nlm.nih.gov/geo/query/acc.cgi=GSE121211, https://www.ncbi.nlm.nih.gov/geo/query/acc.cgi=GSE125779, https://www.ncbi.nlm.nih.gov/geo/query/acc.cgi=GSE108112].
